# Supplementary material for: Integrating cryo-OrbiSIMS with computational modelling and metadynamics simulations enhances RNA structure prediction at atomic resolution
Source: Nat Commun. 2024 May 22;15:4367. doi: 10.1038/s41467-024-48694-3 (PMC11111741; doi:10.1038/s41467-024-48694-3)
Supplement: Supplementary file 1 — Supplementary Information [file 41467_2024_48694_MOESM1_ESM.pdf]

Integrating Cryo-OrbiSIMS with Computational Modelling and Metadynamics Simulations Enhances RNA Structure Prediction at Atomic Resolution

Shannon Ward[1,2,†], Alex Childs[1,2,†], Ceri Staley[1,†], Christopher Waugh[1,2,3], Julie A Watts[4], Anna M Kotowska[4], Rahul Bhosale[6], Aditi N Borkar[1,2,3,*]

Document Contents:

Supplementary Figures 1 to 18

Supplementary Tables 1 to 3

Supplementary Notes 1 to 2

Supplementary Figure 1: (a) Comparison of OrbiSIMS positive polarity spectrum of native bacterial ribosomes under room temperature and cryogenic conditions. Peak intensity for fragments more than 500 m/z units in size is multiplied by 10 and that for fragments more than 1500 m/z units in size is multiplied by 100 for better visualization. (b) Hierarchical clustering of positive polarity cryo-OrbiSIMS data shows that cryo-OrbiSIMS can distinguish the Cas9:sg samples from the ribosomal samples.

(a)

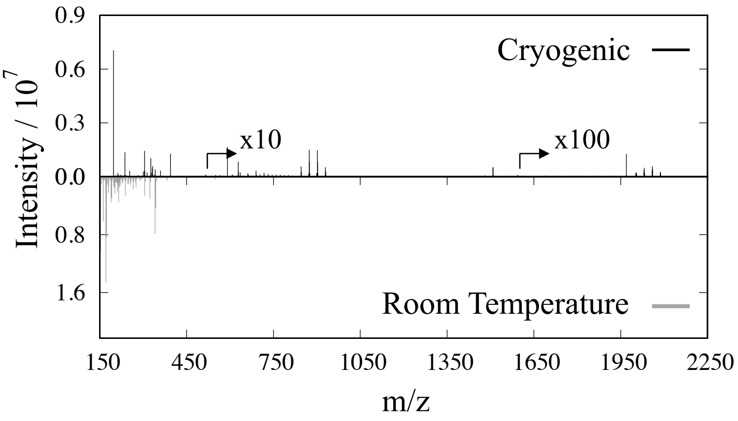

(b)

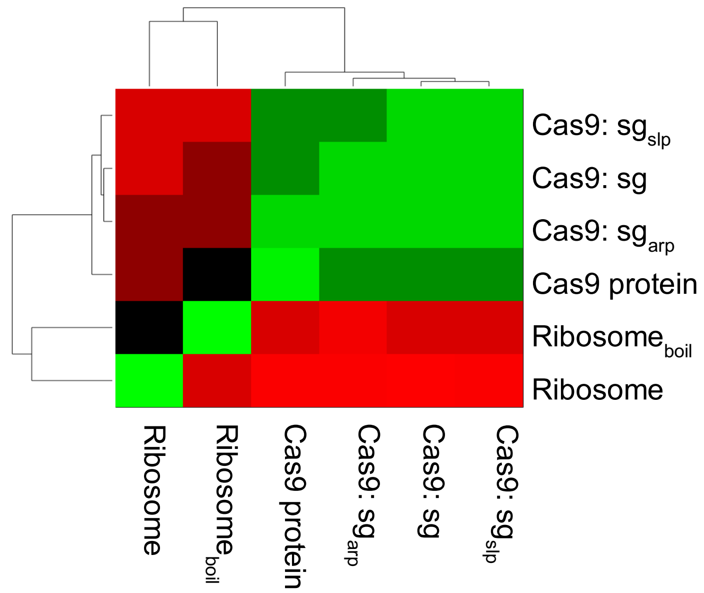

Supplementary Figure 2: Hierarchical clustering of Wasserstein distances between technical repeats of negative polarity data. Replicate number for each sample is denoted in brackets. At 1% significance level, all technical repeats for a given sample are indicated to be drawn from the same distribution.

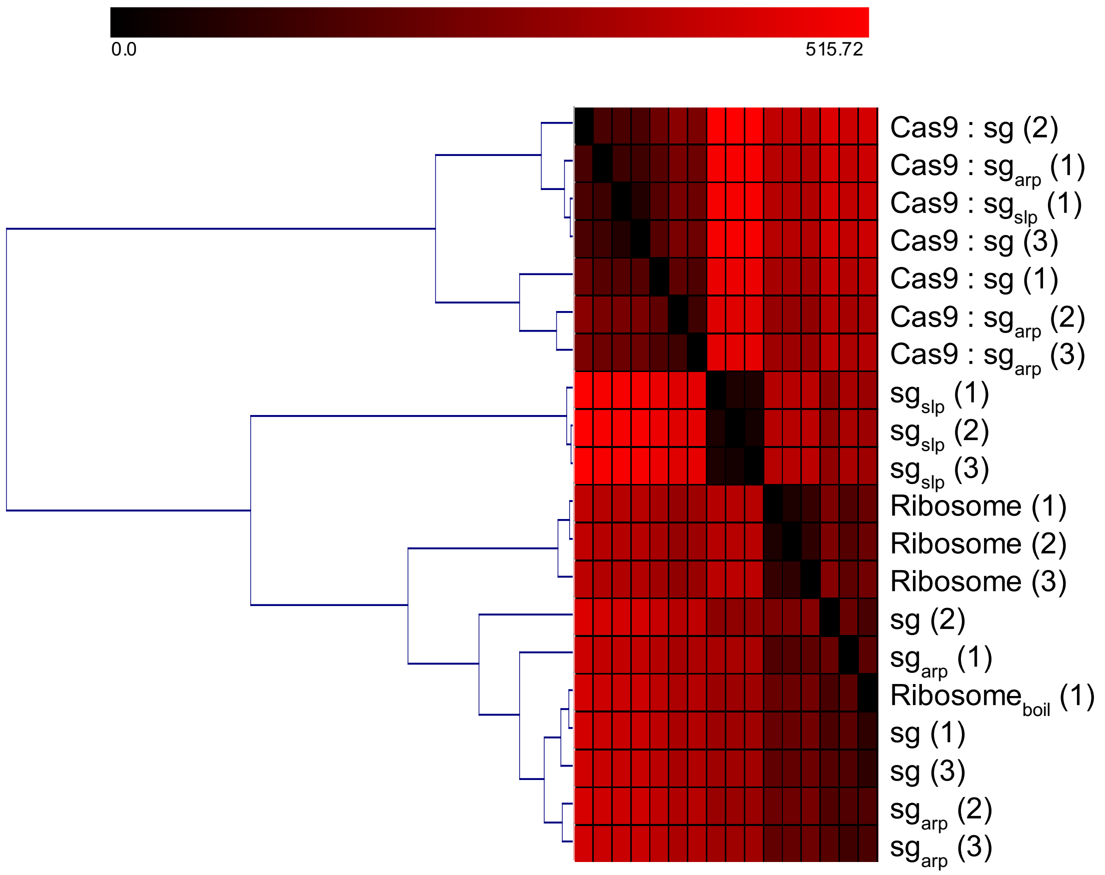

Supplementary Figure 3: Hierarchical clustering of Wasserstein distances between technical repeats of positive polarity data. Replicate number for each sample is denoted in brackets. At 1% significance level, all technical repeats for a given sample are indicated to be drawn from the same distribution.

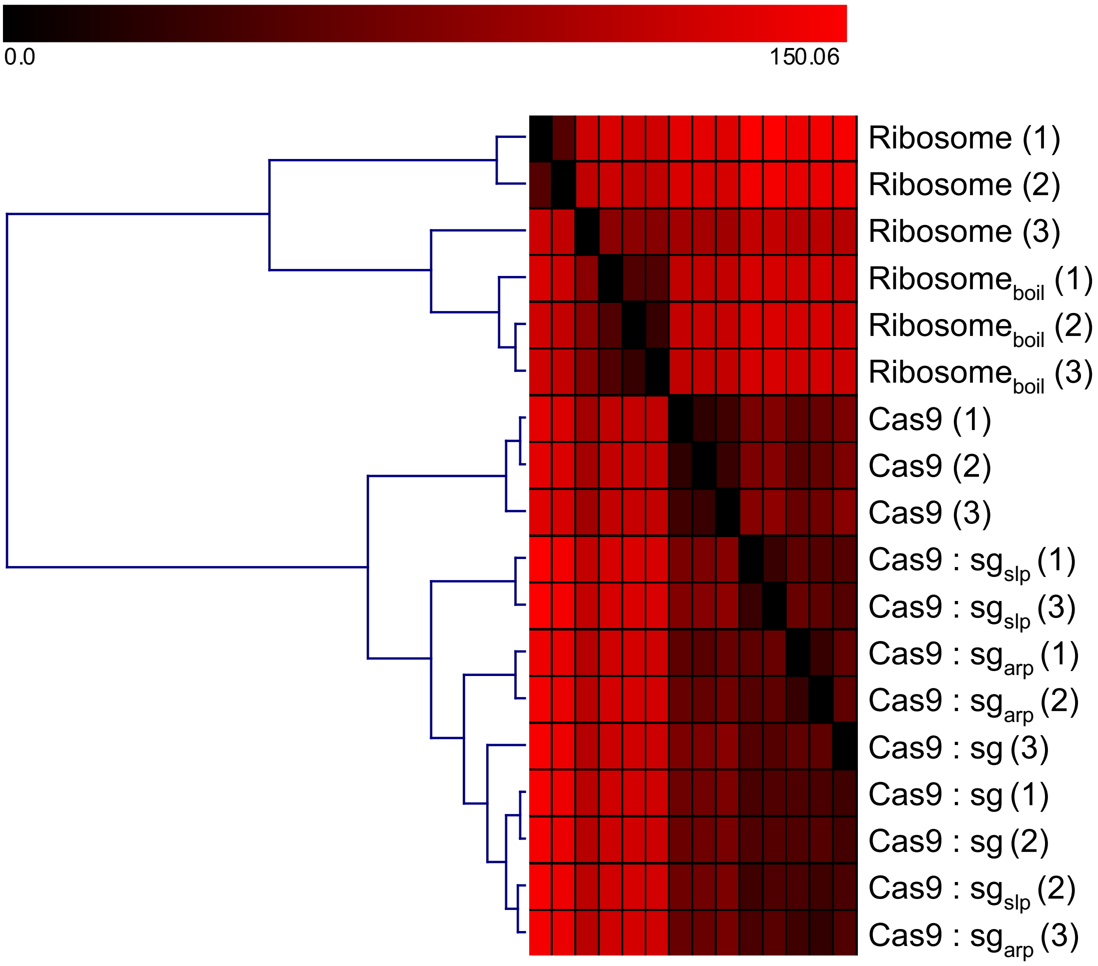

Supplementary Figure 4: Cryo-OrbiSIMS peak assignment statistics for RNA residues in the native Cas9:sg RNP, 7SK RNP and ribosome samples. Magenta panel shows the distribution of RMSE values for all the sliding window lengths used for the assignments and the green panel shows the corresponding distribution of the average assignment redundancy values. The red dot on the green box-plot represents the redundancy value for the lowest RMSE assignment. Dashed black line indicates the average redundancy of 8.13 observed from the RNA sequences from structures deposited in the PDB.

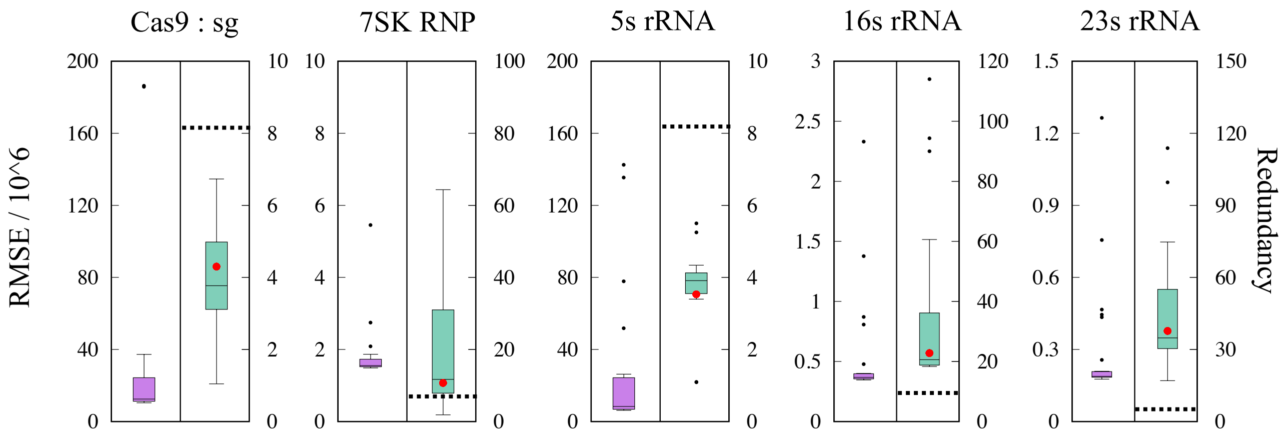

Supplementary Figure 5: Relationships between frequency of cryo-OrbiSIMS data assignment of RNA residues and their SASA values and native contacts within the respective RNA-protein complex. (a-b,e-f) Values for sg RNA residues within the native Cas9:sg RNP complex and (c-d),g-h values for 5s rRNA within the native 70s ribosomes. Figures e-h are scatter plots of the assignment frequencies v/s SASA / native contacts of the residues in the sg or 5s rRNA.

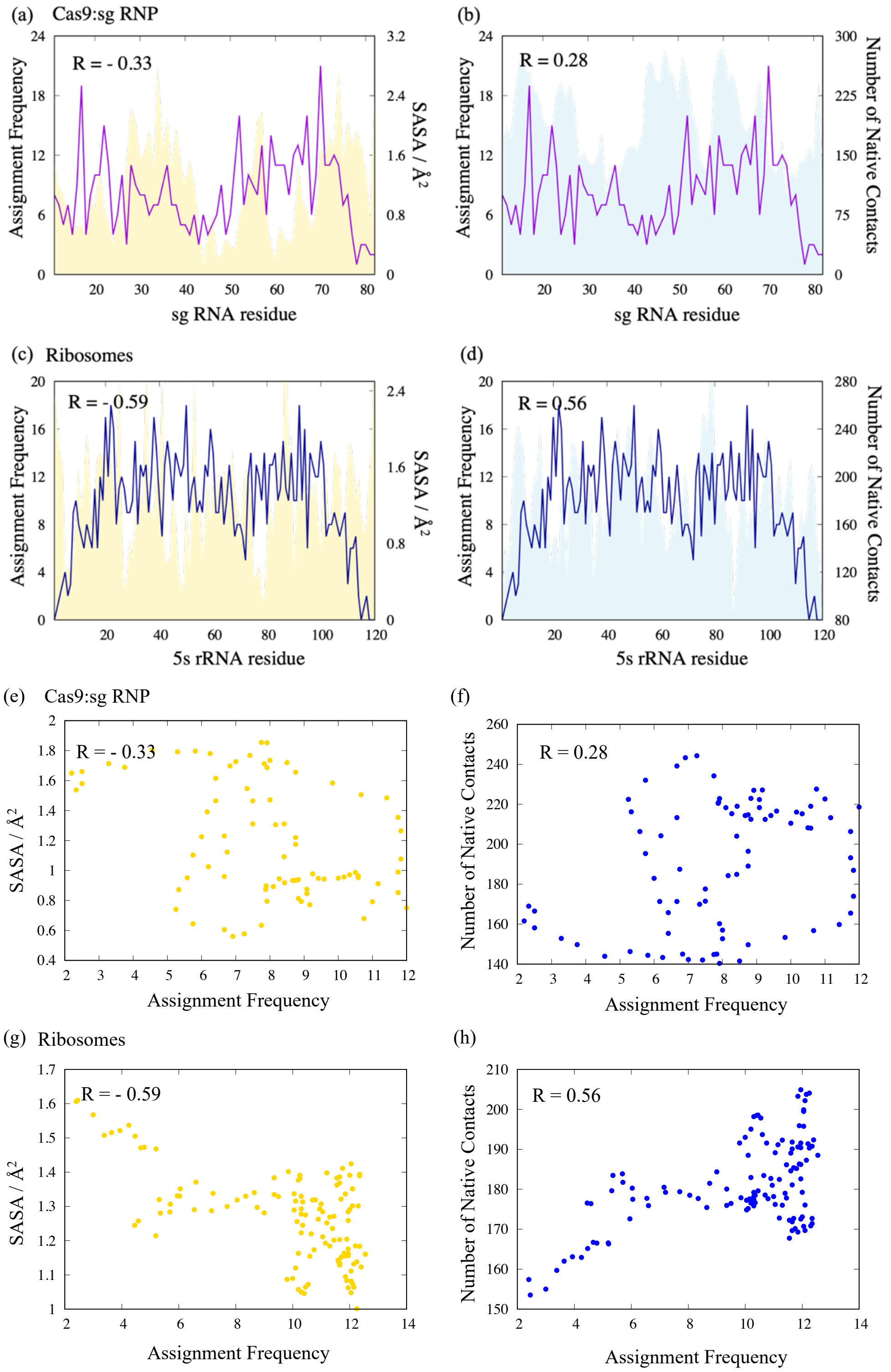

Supplementary Figure 6: 2D structure prediction for the sg RNA in the native Cas9-sg complex.

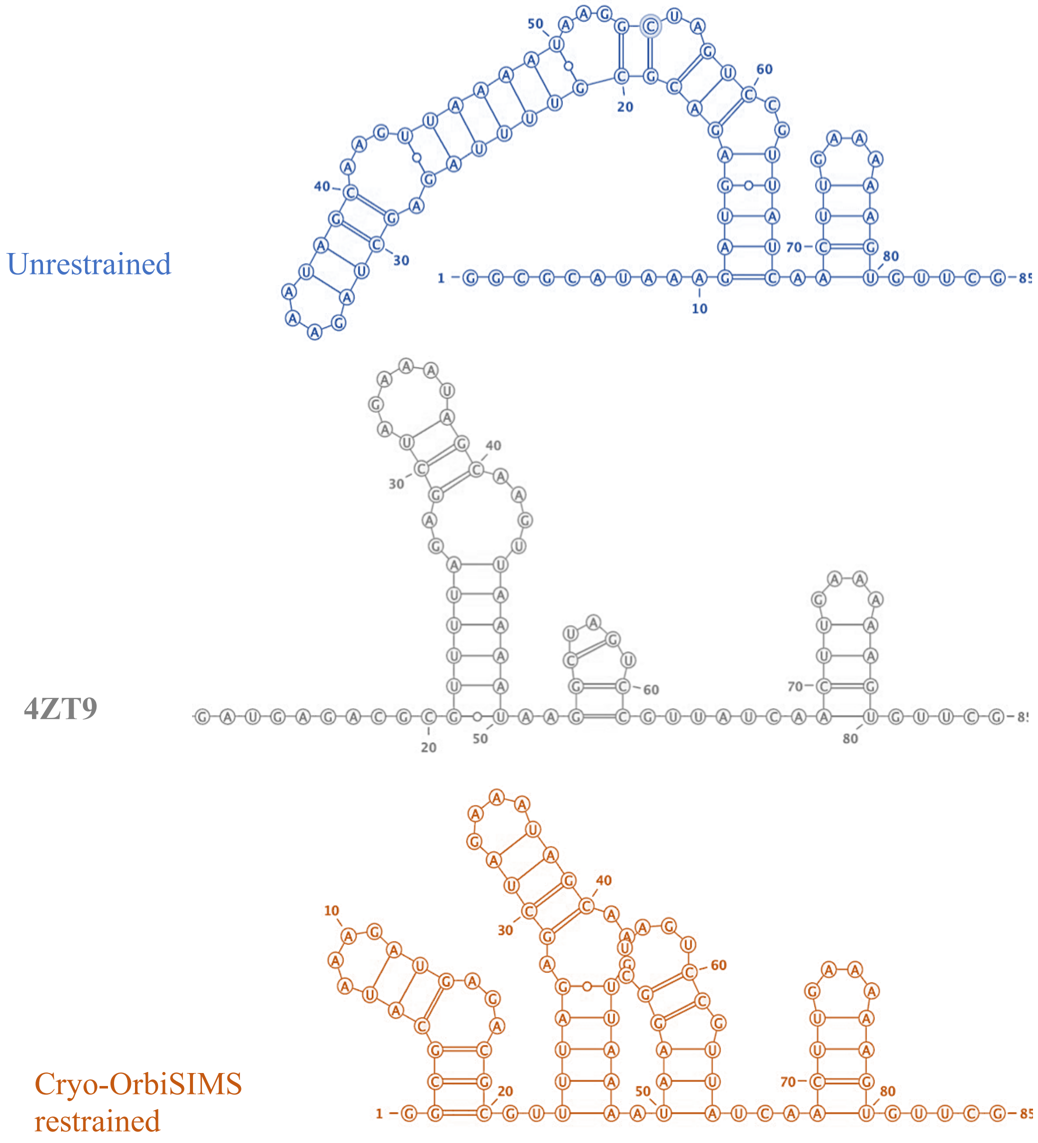

Supplementary Figure 7: 2D structure prediction for the 5s rRNA in the native ribosomal complex.

Unrestrained

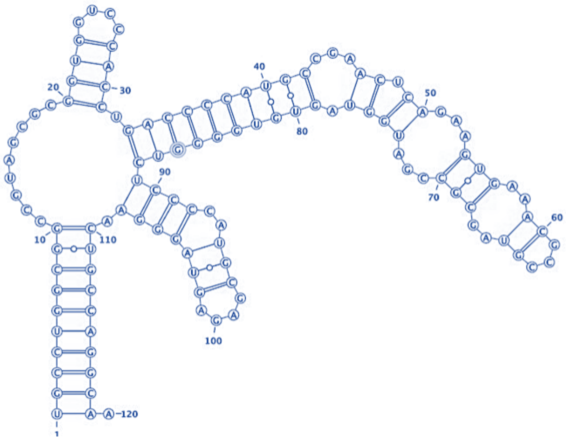

6GWT

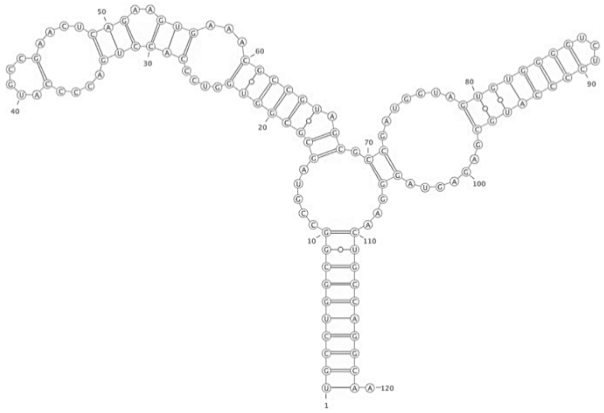

Cryo-OrbiSIMS restrained

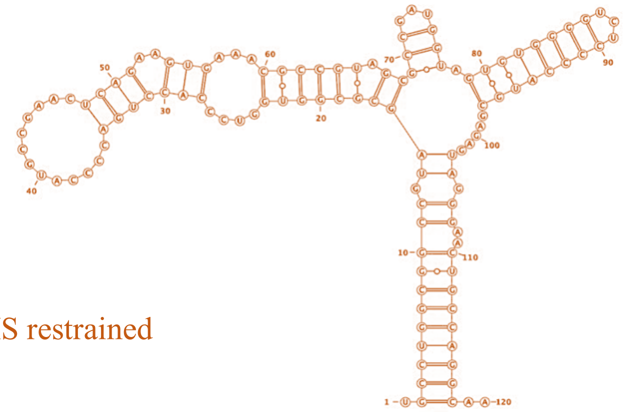

Supplementary Information 8: Comparison of the sg RNA PDB structure with the cryo-OrbiSIMS restrained model of the RNA (a) before and (b) after refinement by metadynamics simulations. In the unrefined model, stemloops 1-2 are flipped by 180° in orientation compared to the repeat – antirepeat stemloop. This orientation gets refined and converges to the native PDB structure during metadynamics simulations.

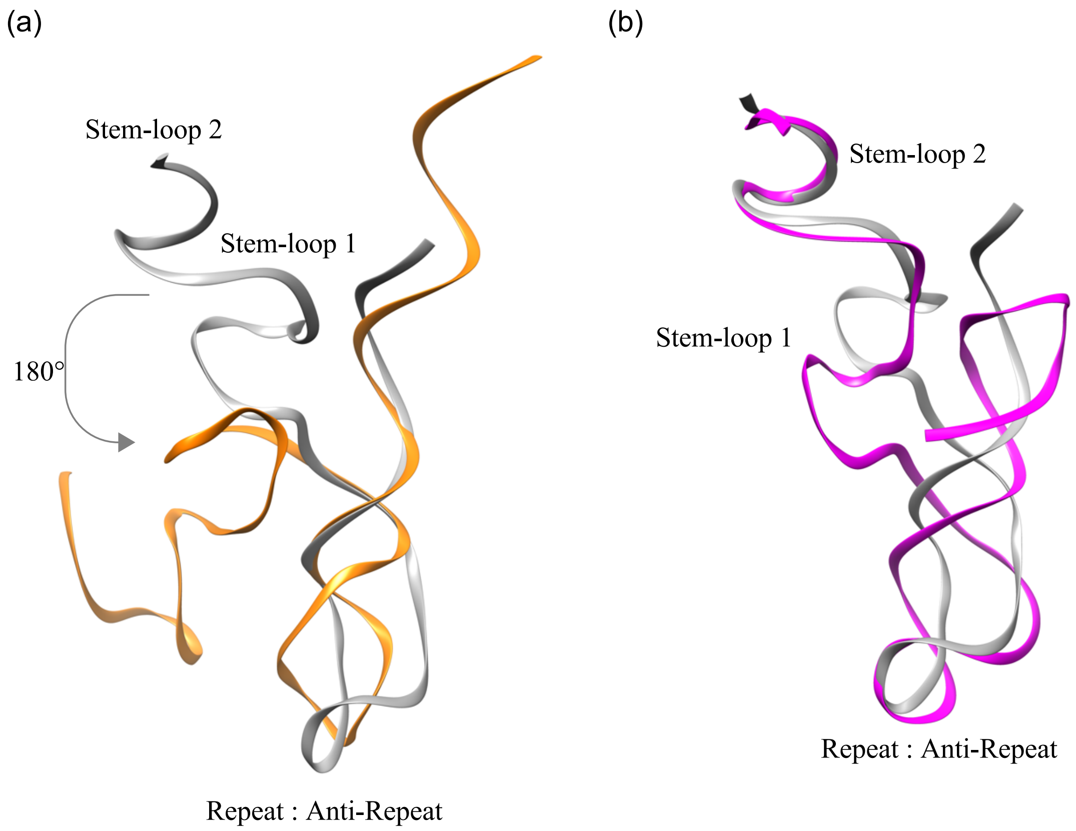

Supplementary Figure 9: Comparison of the 5s rRNA PDB structure with the cryo-OrbiSIMS restrained model of the RNA (a) before and (b) after refinement by metadynamics simulations. In the unrefined model, Loop C is flipped by 180° in orientation and is modelled proximal to Loop D. This orientation gets a bit refined and converges towards the native PDB structure during metadynamics simulations.

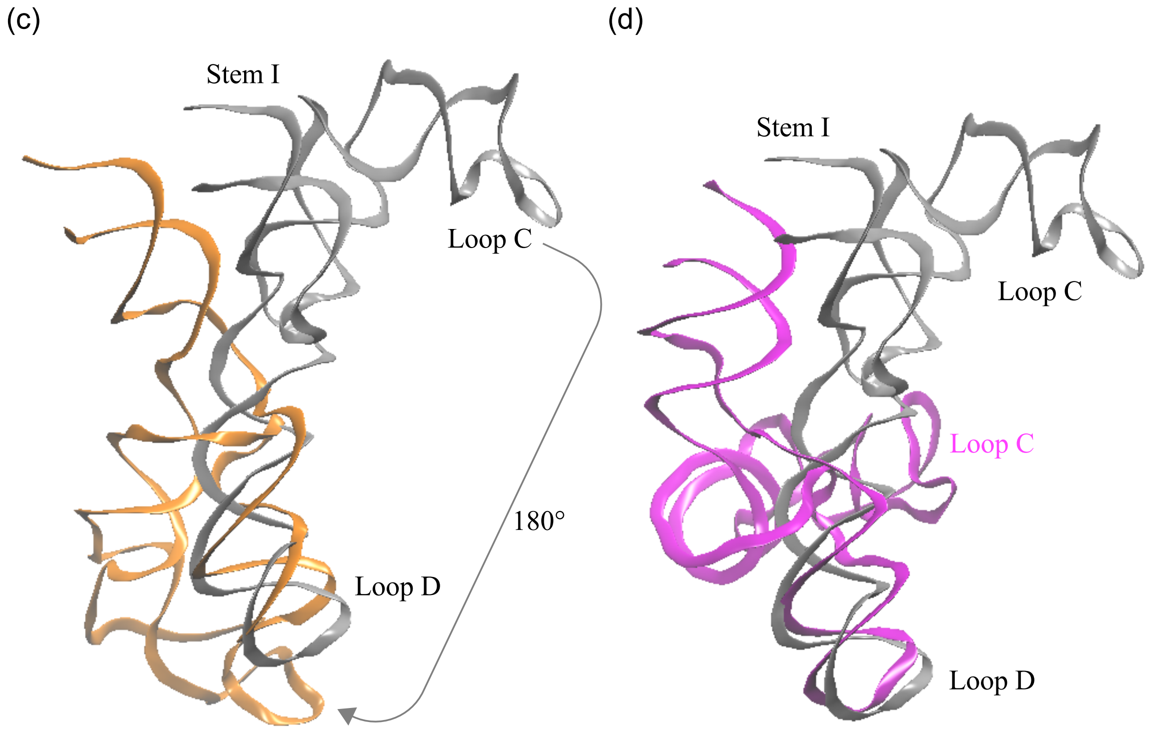

Supplementary Information 10: Free energy landscape derived from the metadynamics simulation of the unrestrained 3D structure for the sg RNA. Unlike the simulations starting from the cryo-OrbiSIMS restrained model, these simulations did not contain well-formed minima and were not able to converge to the PDB structure.

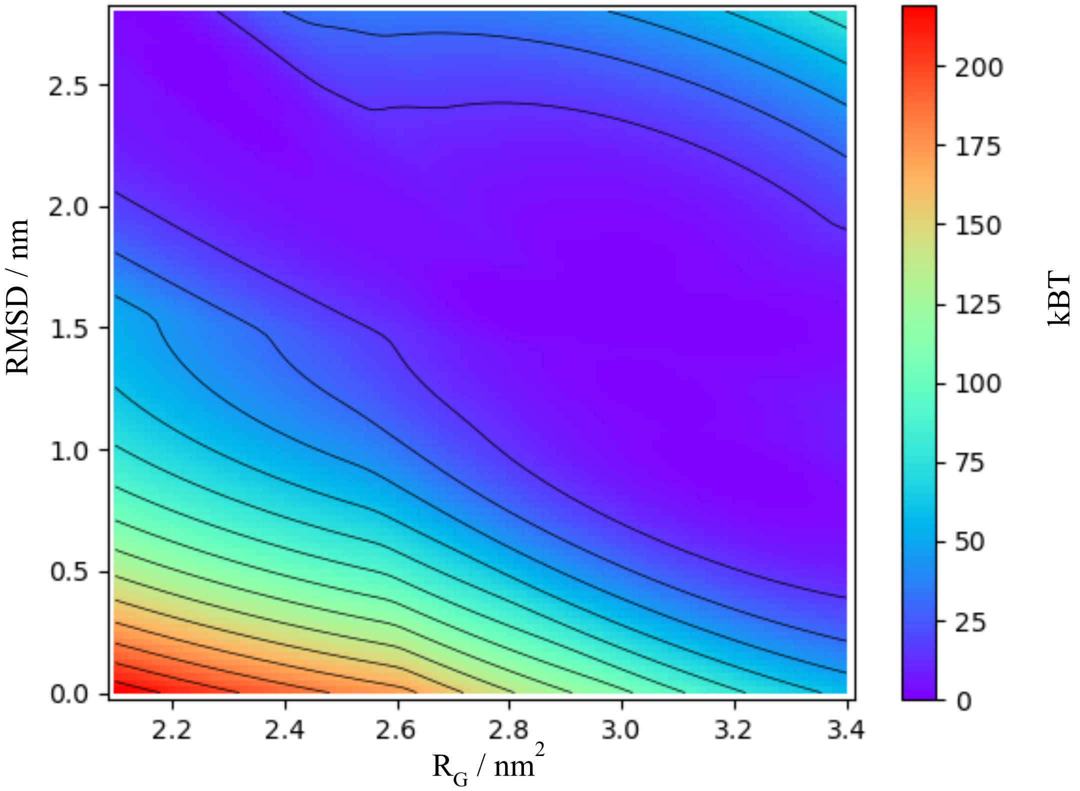

Supplementary Figure 11: (a) Hierarchical clustering and (b) heatmap of percentage similarity between the secondary structures of the 7SK modelled in our study and those previous determined by DMS and SHAPE reactivity.

(a)

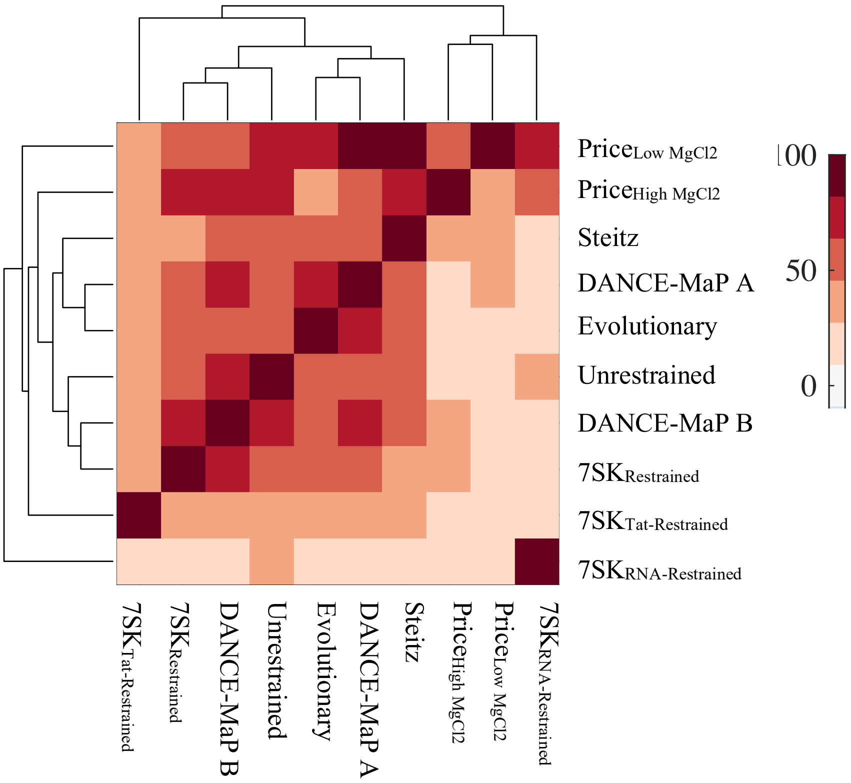

(b)

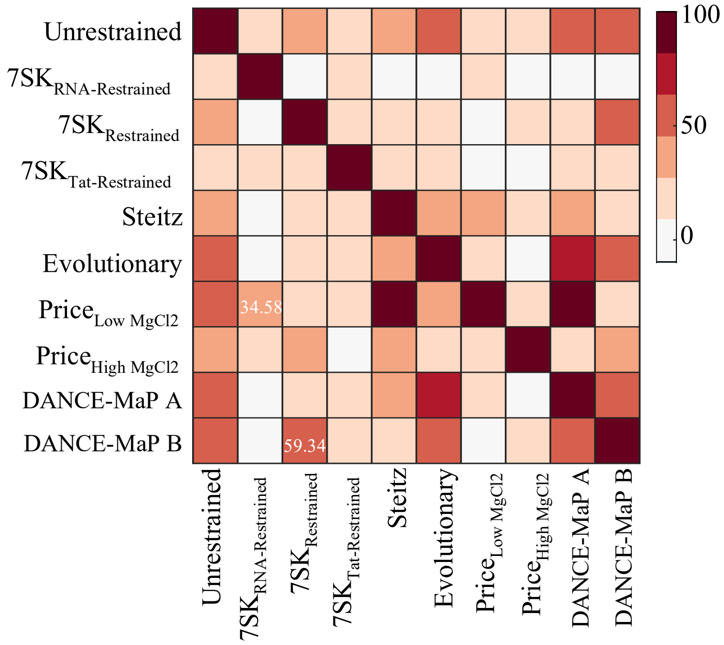

Supplementary Figure 12: Characteristics of RNA sequences within the RNA-protein complex structures deposited in the PDB. (a) Distribution of RNA sequence lengths (magenta histograms) for systems retrieved from the PDB shows a multimodal trend. (b) Relationship between the longest RNA parent sequence that a child fragment of a given length will uniquely align to. The inset shows a comparison of the theoretical limit (gray) for a child fragment of a given length v/s the limit observed from native RNA sequences in the PDB structure database. A seven-nucleotide child fragment is seen to align to its parent sequence as long as 1870 nucleotides. Sequences of up to this length cover more than 94% (gold filled area in (a)) entries in the PDB.

(a)

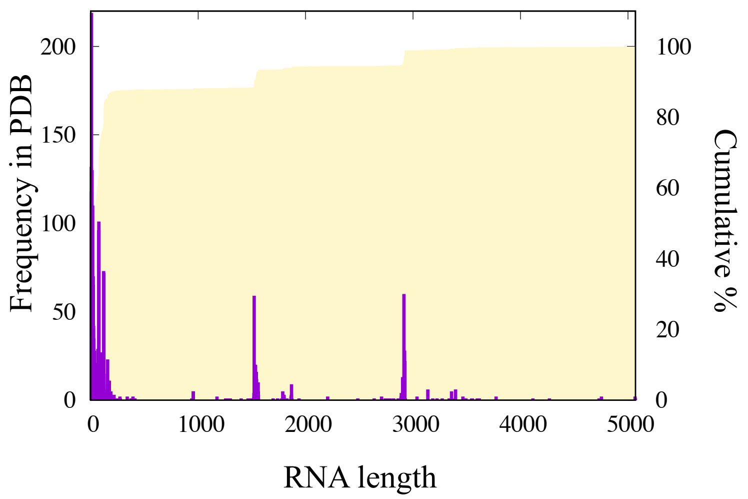

(b)

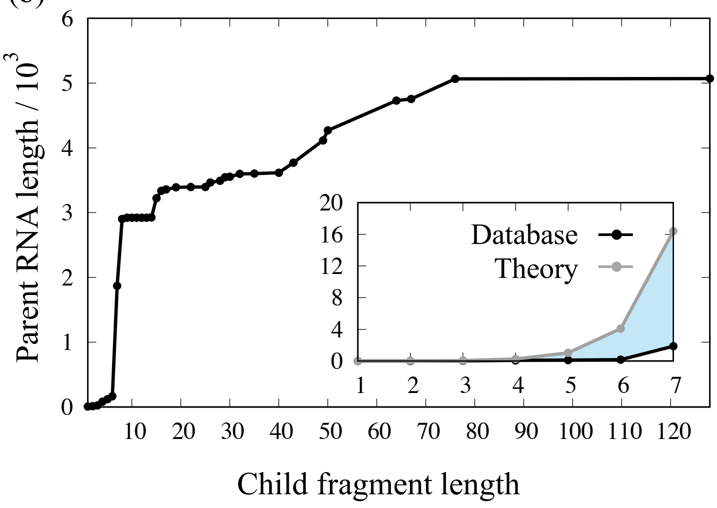

Supplementary Figure 13: Native bacterial ribosome purity and heterogeneity ascertained via negative stain TEM at 80x magnification. Four Micrographs were collected each on 3 replicate samples.

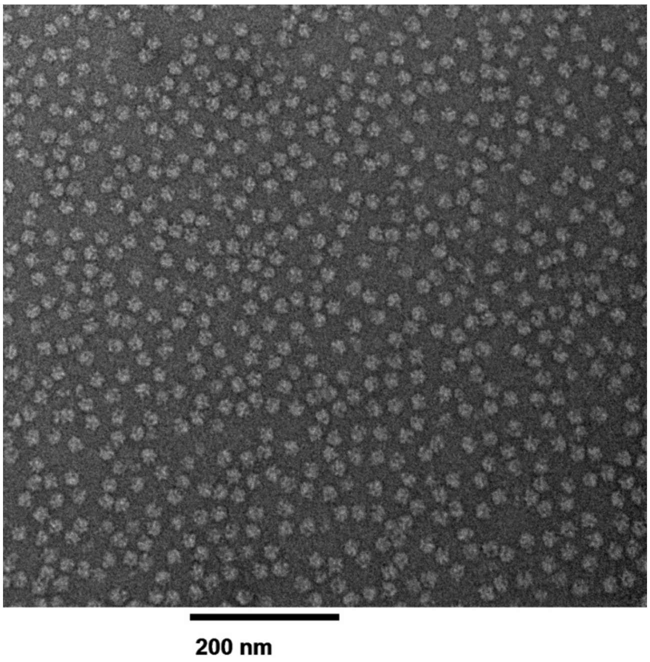

Supplementary Figure 14: Single Guide (sg) RNA sequence from PDB ID 4ZT9 and mutation design. (a) sg RNA mutants to destabilise the repeat : antirepeat interaction and stem-loop 1 structures. Both (c-d) mutants exhibited tertiary structures distinct from the (b) canonical sg RNA structure as predicted via RNAfold and RNAcomposer (webserver.

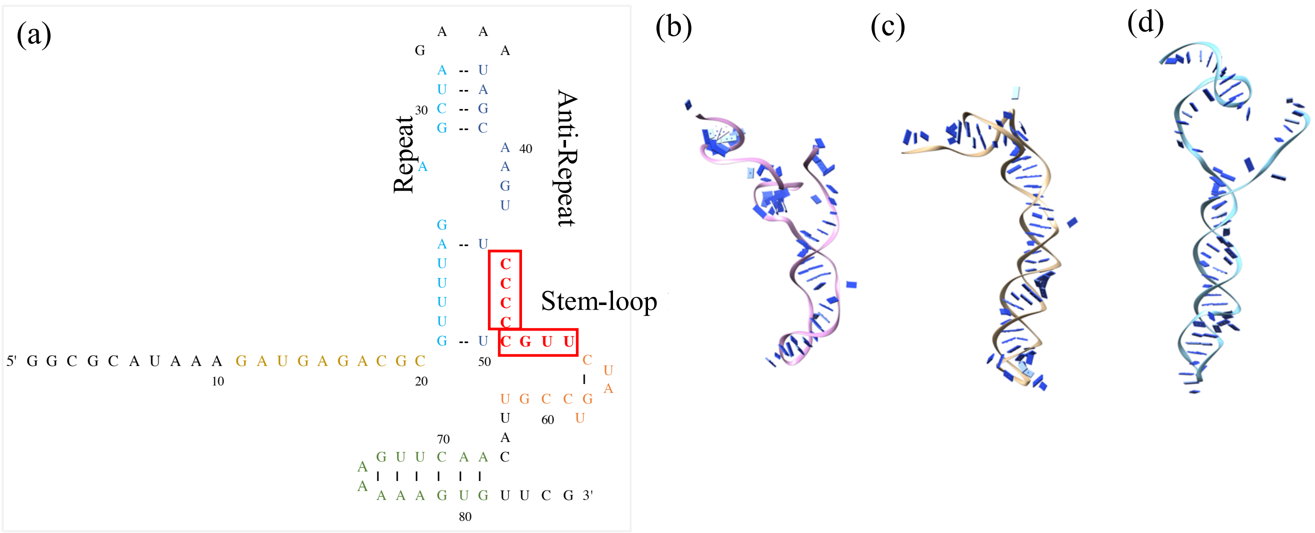

Supplementary Figure 15: Sample composition of native 7SK RNP determined by Western blotting, Mass Spectrometry and reverse transcription PCR. (a) Schematic respresentation of the complex. (b-c) Western blot analysis of 7SK RNP obtained from HeLa cell lysate by (b) co-immunoprecipitation with anti-HEXIM antibody and (c) biotinylated DNA primers complementary to the 7SK RNA. HEXIM-1 (red arrowheads) and CDK9 (yellow arrowheads) were probed in the pull-down samples to confirm the presence of the intact 7SK RNP. Anti-HSP90 pulldown was used a negative control. (d) Volcano plot of the Mass Spectrometry results confirms that the core 7SK RNP proteins are significantly enriched in the pull-down samples compared to controls. (e) Reverse transcription PCR confirms that the full length 7SK RNA is present in the pull-down samples.

(a)

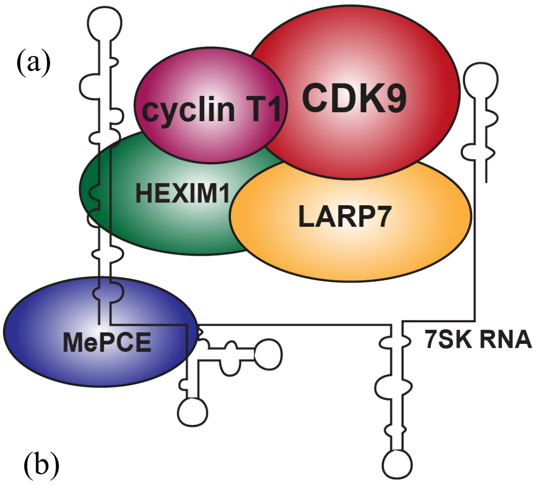

M	Marker
E	Elute
U	Unbound fraction
L	Lysate
W	Washes
B	Boiled beads

(b)

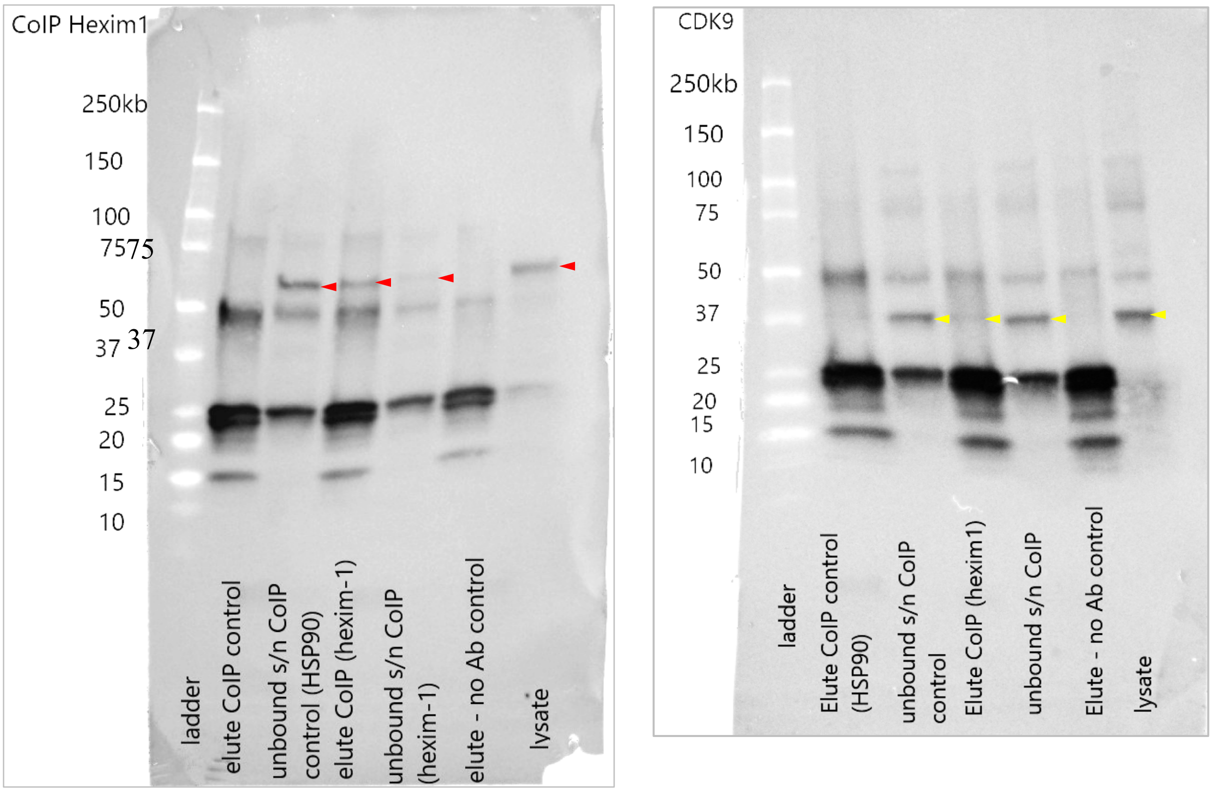

(c)

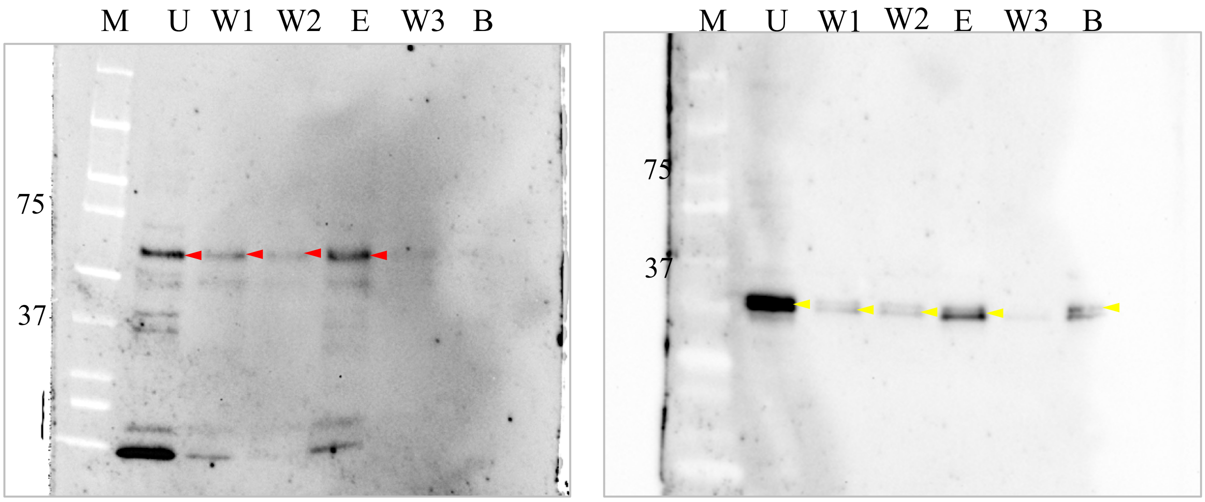
M U W1 W2 E W3 B
75
37
M U W1 W2 E W3 B
75
37

(d)

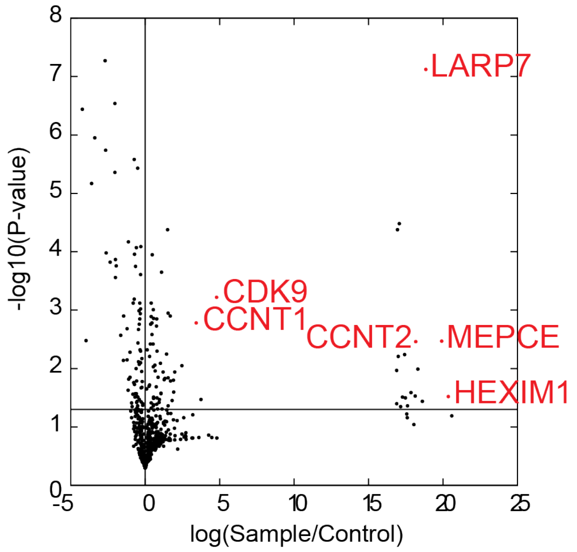
LARP7
CDK9
CCNT1
CCNT2
MEPCE
HEXIM1
-log10(P-value)
log(Sample/Control)
8
7
6
5
4
3
2
1
0
-5
0
5
10
15
20
25

(e)

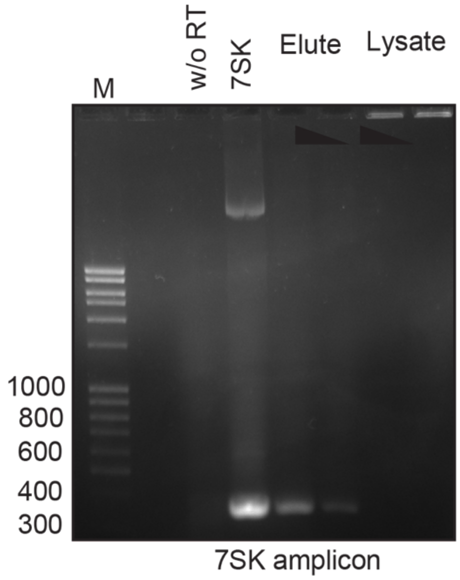
M
w/o RT
7SK
Elute
Lysate
1000
800
600
400
300
7SK amplicon

Supplementary Figure 16: cryo-OrbiSIMS depth profile through the apo 7SK RNA sample as a representative sample. The intensity of RNA peaks (black) rapidly decrease during the data collection and the whole sample is sputtered away at approximately scan number 5. Au^- (yellow), AuS^- (red) and Au_3^- (grey) are also shown for contrast. The intensities for all RNA peaks are summed up at each scan number for better visibility and the Au_3^- ion is presented on a secondary intensity scale for clarity.

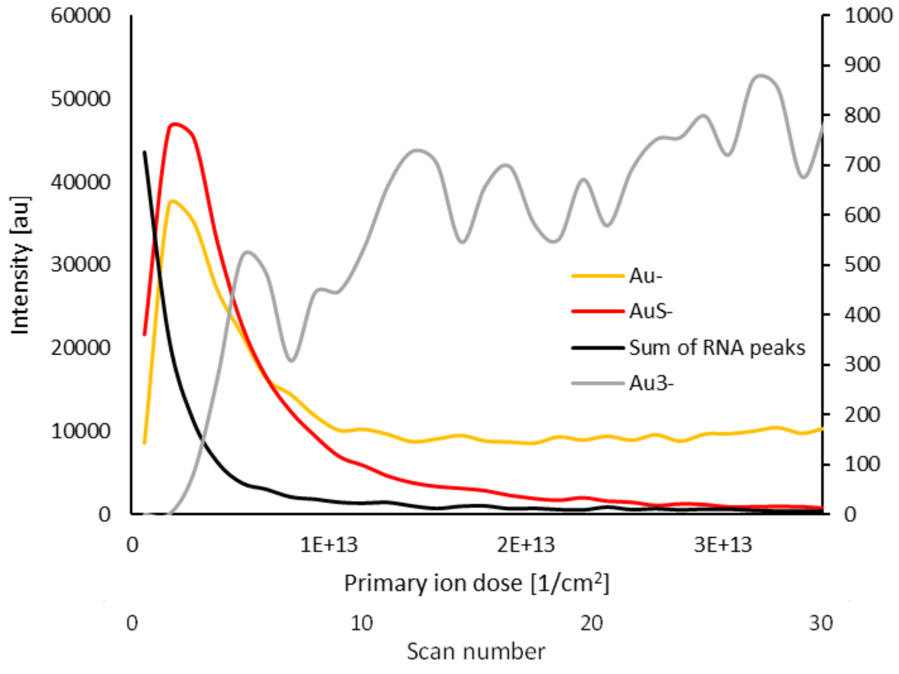

Supplementary Figure 17: Formulating the working hypothesis for cryo-OrbiSIMS induced fragmentation of RNA systems. We present four scenarios (A-D) with a schematic representation of the expected fragmentation pattern that would fit the scenario hypothesis. Scenarios A-C seem to be unlikely as they do not rationalise the observed experimental data. Scenario D aligns with our observations and we thus propose this as a working hypothesis for the cryo-OrbiSIMS induced fragmentation of the RNA-protein systems studied in the this work. Illustration created using Biorender.

Scenarios | **Schematic Fragmentation** Exposed regions: orange; Buried regions: Dark brown | **Hypothesis**

(A)

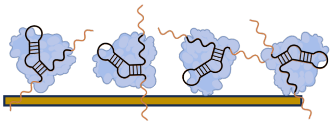

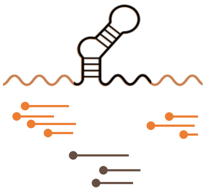

Initial: Exposed regions of RNA would be bombarded more and fragmented more. Thus, more residues from exposed regions will be assigned in the spectrum (more orange fragments).
However, we do not see positive correlation between SASA and peak assignment frequencies in the data.

(B)

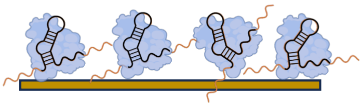

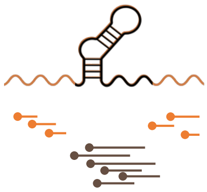

Alternative 1: Molecules preferentially oriented in a monolayer, thus exposed regions would be fragmented more, producing smaller fragments than those produced by inner regions (shorter orange and longer brown fragments).
However, we do not see positive correlation between SASA (or native contacts) with the average fragment length. Thus, molecules are likely randomly oriented on the surface.

(C)

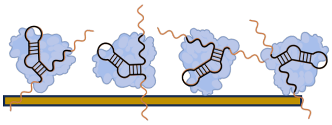

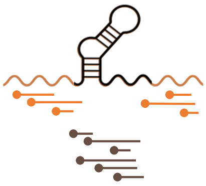

Alternative 2: Molecules randomly oriented in a monolayer and full-depth sputtering achieved by the beam. Thus, whole sample exposed to the beam as some point, so all fragments from all regions should be visible in the spectrum (equal number of orange and brown fragments).
However, we clearly see enrichment from residues from the RNA/protein bound regions in the spectrum

(D)

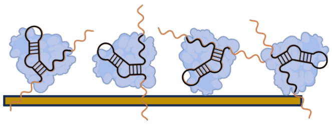

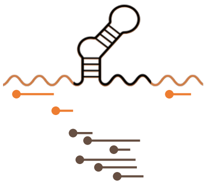

Working model: Molecules randomly oriented as monolayer and full-depth sputtering achieved by the beam. Secondary ion yield is higher from the cohesive regions of the sample exhibiting softer interactions, e.g. the RNA:RNA and RNA:Protein interaction sites (higher number of brown than orange fragments, irrespective of fragment length). This leads to spectral enrichment for the residues from these inner regions, which would now explain the positive correlation between peak assignment frequencies in the spectrum and the number of native contacts of the residues.

Supplementary Figure 18: RNA ion types, numbering and m/z values of single nucleotides. Ion types a-d are described from 5’ end of the sequence and w-z are described from 3’ end. For a fragment of ‘n’ nucleotides in length, there are n-1 types of a-d and w-z ions. The full length fragment ion is denote by M.

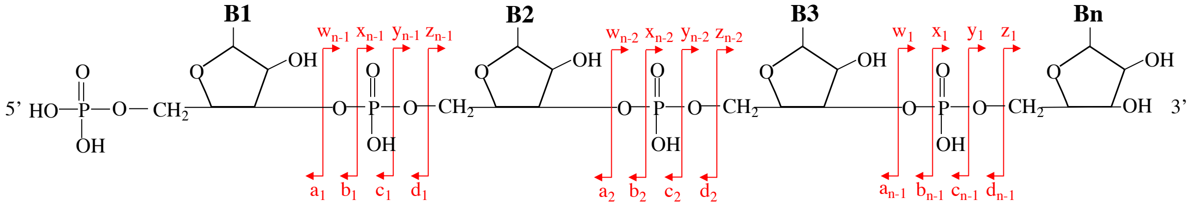

Base	a-B	a1	b1	c1	d1
**A**	193.9980	329.0525	347.0631	409.0189	427.0294
**G**	193.9980	345.0474	363.0580	425.0138	443.0243
**C**	193.9980	305.0413	323.0519	385.0076	403.0182
**U**	193.9980	306.0253	324.0359	385.9916	404.0022

Base	w1	x1	y1	z1
**A**	347.0631	329.0525	267.0968	249.0862
**G**	363.0580	345.0474	283.0917	265.0811
**C**	323.0519	305.0413	243.0855	225.0750
**U**	324.0359	306.0253	244.0695	226.0590

Supplementary Tables

Supplementary Table 1: RNA systems investigated for benchmarking cryo-OrbiSIMS

No.	Sample	Description
1	Cas9	Native Protein
2	Cas9 : sg	Native RNA – protein complex, wildtype sg RNA
3	Cas9 : sg_{slp}	Native RNA – protein complex, mutant sg RNA, $A_{51}A_{52}G_{53}G_{54}$ to $C_{51}G_{52}U_{53}U_{54}$
4	Cas9 : sg_{arp}	Native RNA – protein complex, mutant sg RNA, $A_{46}A_{47}A_{48}A_{49}$ to $C_{46}C_{47}C_{48}C_{49}$
5	sg RNA	Apo RNA, wildtype
6	sg_{slp} RNA	Apo RNA, $A_{51}A_{52}G_{53}G_{54}$ to $C_{51}G_{52}U_{53}U_{54}$ mutant
7	sg_{arp} RNA	Apo RNA, $A_{46}A_{47}A_{48}A_{49}$ to $C_{46}C_{47}C_{48}C_{49}$ mutant
8	Ribosome	Natively purified bacterial ribosome
9	$Ribosome_{boiled}$	Denatured ribosomes obtained by boiling the native sample

Supplementary Table 2: Comparison between the cryo-OrbiSIMS restrained RNA 2D structures with the previously established 2D structures determined using chemical probing restraints. The table includes comparisons between (A) secondary structure assigned from PDB 4ZT9 and 6GWT by RNApdbee for the sg RNA and 5s rRNA respectively; (B) secondary structure derived from cryo-OrbiSIMS; (C) secondary structure derived from SHAPE-MaP or equivalent chemical probing technique where available and (D) secondary structure predicted by RNAstructure. The similarities (in terms of F1-score) are indicated between A and B, between A and C, between A and D, and between B and C. The comparison between cryo-OrbiSIMS restrained 2D structures of 5s rRNA and 7SK RNA with their respective chemically probed structures, indicate strong complementary between the structural readout of the two techniques.

System	PDB with cryo-OrbiSIMS (A and B)	PDB with SHAPE-MaP (A and C)	PDB with RNAstructure (A and D)	Cryo-OrbiSIMS with SHAPE-MaP (B and C)
**Cas9-sg RNP complex**	0.7	-	0.63	-
**5s rRNA in bacterial ribosomal complex**	0.825	0.71*<br>0.84**	0.38	0.69*<br>0.98**
**7SK in native RNP**	-	-	-	0.82[31]

*DMS probing[20,21] (*in vivo*)

**IM7 probing [5SRRNA_IM7_0006[22], *in vivo*. where the chemical probing was performed on extracted RNA (e.g. 5SRRNA_IM7_0009[22,23]), the F1-score was 0.34. However, this latter experimental condition is not equivalent to our cryo-OrbiSIMS samples conditions, nor to the PDB structure, and thus the value was omitted from the comparison table above]

Supplementary Table 3: (a) RMSD values (in Å) and TM-Scores to assess the structural similarity between the modelled tertiary structures and the PDB structures of the sg RNA and the 5s rRNA. The table denotes comparisons between the (A) experimentally determined PDB structures (4ZT9 and 6GWT for the Cas9-sg and 5s RNA respectively) with the corresponding RNA structures derived from either the (B) 2D folds extracted from the PDB structure using RNApdbee, (C) 2D folds predicted from RNA sequence alone, or (D) cryo-OrbiSIMS restrained 2D folds. Finally, a comparison between the PDB structures and (E) the metadynamics refined structures is also included. The TM-score values are calculated using from US-align, which performs three-dimensional structure alignments for monomeric and complex protein and nucleic acid structures using the TM-score and heuristic structural alignment algorithms. The results indicate that the higher global RMSD values of the predicted structures (A v/s B, A v/s C and A v/s D) cannot necesarrily be attributed to any inefficiency of the 3D prediction algorithm. However, using the cryo-OrbiSIMS restrained 2D folds (A v/s D) and further 3D structure refinement using metadynamics (A v/s E) leads to significant improvement in the TM-score values compared to when no restraints are used (A v/s C), indicating a higher similarity of the restrained structures compared to the PDB structuresWe propose that this improvement for the refined structures is particularly significant because it is higher than the mean TM-Score of 0.273 calculated for pairwise RNA structure alignment of n = 168,917 chain pairs derived from the PDB.

System	A v/s B		A v/s C		A v/s D		A v/s E	
	RMSD	TM-Score	RMSD	TM-Score	RMSD	TM-Score	RMSD	TM-Score
sg RNA in Cas9:sg RNP	17.9	0.1965	15.8	0.1887	23.3	0.2218	7.8	0.2943
5s rRNA in native Ribosomes	3.2	0.6525	39.4	0.1883	28.3	0.2833	20.7	0.396

Supplementary Notes

Supplementary Note 1: Comparison of SHAPE-MaP technique with Cryo-OrbiSIMS for secondary structure determination of RNA

SHAPE-MaP and cryo-OrbiSIMS are two independent techniques that can be used for RNA secondary structure determination. SHAPE-MaP is more established, while cryo-OrbiSIMS requires specialist instrumentation and expertise in data interpretation that isn't yet widely available. We have provided a brief comparison of the techniques in the table below.

	**SHAPE-MaP**	**Cryo-OrbiSIMS**
**Description**	Combination of SHAPE-Seq and mutational profiling techniques	Mass spectrometry technique that analyses fragments generated through Secondary Ion Mass Spectrometry
**Sample requirement**	Microgram quantities of RNA / sample	Pico - nanomolar quantities of sample
**RNA length**	No size limitation, although the PCR amplification step and library preparation for sequencing could impose a lower limit to length reliably probed by the technique.	No size limitation on the size of RNA probed. However, the redundancy in peak assignments progressively increases with the length of the RNA studied. Thus, the confidence in assignments could get worse for RNAs longer than a few hundred nucelotides.
**Resolution**	Nucleotide level	Nucleotide level
**Experimental read-out**	The rate of the chemical modification provides information about the flexibility of RNA	The frequency of peak assignments for each residue provides information about its native contacts.
**Time**	Technique involves multiple steps, which can take considerable for data acquisition and analysis.	For thin-layer samples, data acquisition completed within minutes. Automated data analysis requires a few hours to days.
**Skills**	Skilled personnel for sample preparation, chemical modification, sequencing and data analysis	Expertise in instrument operation required

**Instrumentation and availability**	Widely adopted technique with specialist data analysis software available for data interpretation.	Mass spectrometry instruments can be expensive, and OrbiSIMS requires specialist instrumentation and expertise in data interpretation that isn't yet widely available.

Supplementary Note 2: Complementarity of LC-MS and Cryo-Electron Microscopy with Cryo-OrbiSIMS

Liquid Chromatography-Mass Spectrometry (LC-MS) and Cryo-OrbiSIMS are analytical techniques used in different contexts for biomolecular investigations. LC-MS is commonly used in proteomics studies, whereas OrbiSIMS provides spatial information about either the distribution of the analyte or its structural configuration within the sample. Unlike OrbiSIMS, LC-MS is performed on denatured and enzymatically digested samples and is thus not capable of providing information on the native 3D structure of the biomolecules studied.

Cryo-Electron Microscopy on the other hand is a nanoscale imaging technique used to determine the 3D structures of biological macromolecules at high-resolution. However, cryo-EM has stringent sample requirements and thus, cryo-OrbiSIMS could be suitable for investigating the structural properties of conformationally dynamic and heterogeneous samples, such as natively purified complexes at analytical scales or those containing RNAs, where it is challenging to achieve the sample purity, homogeneity and yields required for cryo-EM analysis.

Further comparison of the three techniques is provided in the table below.

	**LC-MS**	**Cryo-OrbiSIMS**	**Cryo-Electron Microscopy**
**Description**	Analytical technique that combines physical separation of analyte using liquid chromatography with mass spectrometry	Mass spectroscopy technique that analyses fragments generated through Secondary Ion Mass Spectrometry	Nanoscale imaging technique
**Biomolecular Applications**	Proteomics	Determining the spatial distribution of analyte *in situ*	Determining the 3D structures of biomolecules
**Sample requirement**	Enzymatically digested samples at nano – micromolar concentrations	Pico - nanomolar quantities of sample	Pure (>95%), stable and homogeneous biomolecular complexes.
**Biomolecular size**	N/A	N/A	Typically, a few hundred kDa in size, although smaller complexes have also been investigated
**Resolution**	Amino-acid / Nucleotide level	Amino-acid / Nucleotide level	Near-atomic
**Experimental read-out (relevant**	Mass / charge ratio of the analyte	The frequency of peak assignments for each	2D Electron Micrographs are

**to the present manuscript)**	fragments are compared against standard databases to identify protein components in the samples.	residue provides information about its native contacts.	processed via single particle analysis to reconstruct the 3D volume of the biomolecules
**Time**	Sample processing, data collection and analysis can be routinely conducted in a few days.	For thin-layer samples, such as the biological monolayers analysed in this work, data acquisition completed within minutes. Automated data analysis can be completed within a few hours to days.	Preparing samples at the required yield, purity and homogeneity and consequent optimisation of grid freezing can be challenging, requiring months to years. Data typically collected over 2-3 days and analysis can take weeks to a few months.
**Skills**	Skilled personnel for sample preparation, instrument handling and data analysis	Expertise in instrument operation required, automated data analysis can be completed by semi-skilled personnel.	Expertise in instrument operation and data analysis required
**Instrumentation and availability**	Although MS instruments are a specialised piece of equipment, LC-MS is a widely adopted technique with specialist data analysis software available for data interpretation.	Mass spectrometry instruments can be expensive, and OrbiSIMS requires specialist instrumentation and expertise in data interpretation, which isn't yet widely available.	High-resolution Cryo-EMs are expensive and require steep maintenance and service contracts. Instrument availability can be a bottleneck, yet the technique has become mainstream for structural analysis of large biomolecular complexes.
