# Supplementary material for: Integrating cryo-OrbiSIMS with computational modelling and metadynamics simulations enhances RNA structure prediction at atomic resolution
Source: Nat Commun. 2024 May 22;15:4367. doi: 10.1038/s41467-024-48694-3 (PMC11111741; doi:10.1038/s41467-024-48694-3)
Supplement: Supplementary file 3 — Description of Additional Supplementary Information [file 41467_2024_48694_MOESM3_ESM.docx]

Description of Additional Supplementary Information

Supplementary Data 1

This file contains the complete cryo-OrbiSIMS peak assignments. Furthermore, it also contains information on the frequency of individual residue assignments and the average length of fragments in which it is assigned.
