# Supplementary material for: Integrating cryo-OrbiSIMS with computational modelling and metadynamics simulations enhances RNA structure prediction at atomic resolution
Source: Nat Commun. 2024 May 22;15:4367. doi: 10.1038/s41467-024-48694-3 (PMC11111741; doi:10.1038/s41467-024-48694-3)
Supplement: Supplementary file 5 — Reporting Summary [file 41467_2024_48694_MOESM5_ESM.pdf]

nature portfolio

Corresponding author(s): Aditi N Borkar

Last updated by author(s): Apr 11, 2024

Reporting Summary

Nature Portfolio wishes to improve the reproducibility of the work that we publish. This form provides structure for consistency and transparency in reporting. For further information on Nature Portfolio policies, see our Editorial Policies and the Editorial Policy Checklist.

Statistics

For all statistical analyses, confirm that the following items are present in the figure legend, table legend, main text, or Methods section.

n/a	Confirmed	
☐	☒	The exact sample size (*n*) for each experimental group/condition, given as a discrete number and unit of measurement
☐	☒	A statement on whether measurements were taken from distinct samples or whether the same sample was measured repeatedly
☐	☒	The statistical test(s) used AND whether they are one- or two-sided<br>*Only common tests should be described solely by name; describe more complex techniques in the Methods section.*
☐	☒	A description of all covariates tested
☒	☐	A description of any assumptions or corrections, such as tests of normality and adjustment for multiple comparisons
☐	☒	A full description of the statistical parameters including central tendency (e.g. means) or other basic estimates (e.g. regression coefficient) AND variation (e.g. standard deviation) or associated estimates of uncertainty (e.g. confidence intervals)
☐	☒	For null hypothesis testing, the test statistic (e.g. *F*, *t*, *r*) with confidence intervals, effect sizes, degrees of freedom and *P* value noted<br>*Give P values as exact values whenever suitable.*
☒	☐	For Bayesian analysis, information on the choice of priors and Markov chain Monte Carlo settings
☒	☐	For hierarchical and complex designs, identification of the appropriate level for tests and full reporting of outcomes
☐	☒	Estimates of effect sizes (e.g. Cohen's *d*, Pearson's *r*), indicating how they were calculated

Our web collection on statistics for biologists contains articles on many of the points above.

Software and code

Policy information about availability of computer code

Data collection	OrbiSIMS spectra were acquired using IonToF SurfaceLab 7.2.128699
Data analysis	Custom scripts written for MATLAB 2021a were used to perform all the data analysis. These script files along with a comprehensive code documentation file that enlists the system requirements, installation instructions, step-by-step instructions for running the scripts and explanations of file formats and functionalities, and a sample dataset for users to test and understand the code's functionality are available on GitHub repository https://github.com/BorkarLab/OrbiSIMS_RNA_analysis/tree/RNA_published60 with DOI 10.5281/zenodo.10960751.

For manuscripts utilizing custom algorithms or software that are central to the research but not yet described in published literature, software must be made available to editors and reviewers. We strongly encourage code deposition in a community repository (e.g. GitHub). See the Nature Portfolio guidelines for submitting code & software for further information.

Data

Policy information about availability of data

All manuscripts must include a data availability statement. This statement should provide the following information, where applicable:

- Accession codes, unique identifiers, or web links for publicly available datasets
- A description of any restrictions on data availability
- For clinical datasets or third party data, please ensure that the statement adheres to our policy

The raw cryo-OrbiSIMS data generated in this study is deposited in The University of Nottingham Collection of Public Research Data59 [http://doi.org/10.17639/

nott.7354] and can also be made available upon request from the corresponding author. All processed data supporting the findings of this study is provided within the main text and supplementary information. The following Protein Data Bank entries were used to benchmark the cryo-OrbiSIMS modelled 2D and 3D structures: (1) PDB ID 4ZT9 for Cas9:sg complex [https://doi.org/10.2210/pdb4ZT9/pdb], (2) PDB ID 6GWT [https://doi.org/10.2210/pdb6GWT/pdb] for bacterial ribosome complex, and (3) PDB IDs 5IEM [https://doi.org/10.2210/pdb5IEM/pdb], 5LYS [https://doi.org/10.2210/pdb5LYS/pdb], 5LYU [https://doi.org/10.2210/pdb5LYU/pdb], 5LYV [https://doi.org/10.2210/pdb5LYV/pdb], 6MCI [https://doi.org/10.2210/pdb6MCI/pdb], 6MCF [https://doi.org/10.2210/pdb6MCF/pdb], 6DCB [https://doi.org/10.2210/pdb6DCB/pdb], 6DCC [https://doi.org/10.2210/pdb6DCC/pdb], 2KX8 [https://doi.org/10.2210/pdb2KX8/pdb], 6D12 [https://doi.org/10.2210/pdb6D12/pdb], 7SLP [https://doi.org/10.2210/pdb7SLP/pdb] and 7SLQ [https://doi.org/10.2210/pdb7SLQ/pdb] for the 7SK RNP complex. Additionally, DMS probing dataset [https://doi.org/10.7554/eLife.22037] and IM7 probing dataset from RMDB 5SRRNA_IM7_0006 [https://rmdb.stanford.edu/detail/5SRRNA_1M7_0006] were used for performance comparison of cryo-OrbiSIMS with these techniques.

Research involving human participants, their data, or biological material

Policy information about studies with human participants or human data. See also policy information about sex, gender (identity/presentation), and sexual orientation and race, ethnicity and racism.

Reporting on sex and gender	The research doesn't involve human participants and thus this information has not been collected.
Reporting on race, ethnicity, or other socially relevant groupings	The research doesn't involve human participants and thus this information has not been collected.
Population characteristics	The research doesn't involve human participants and thus this information has not been collected.
Recruitment	The research doesn't involve human participants and thus this information has not been collected.
Ethics oversight	The research doesn't involve human participants and thus this information has not been collected.

Note that full information on the approval of the study protocol must also be provided in the manuscript.

Field-specific reporting

Please select the one below that is the best fit for your research. If you are not sure, read the appropriate sections before making your selection.

☒ Life sciences ☐ Behavioural & social sciences ☐ Ecological, evolutionary & environmental sciences

For a reference copy of the document with all sections, see nature.com/documents/nr-reporting-summary-flat.pdf

Life sciences study design

All studies must disclose on these points even when the disclosure is negative.

Sample size	Sample size is not applicable to this study as the mass spectrum generated via cryo-OrbiSIMS is dependent upon complex biochemical characteristics of the RNA system studied.
Data exclusions	No data was excluded from this study.
Replication	Three technical replicates were collected for each RNA system studied and under each sample preparation conditions (native, denaturing or apo). Three biological replicates were also collected for the 7SK RNP system. All replication attempts were successful.
Randomization	Randomization is not applicable to this study as the mass spectrum collected for each RNA system is a unique fingerprint and is dependent upon the RNAs biochemical and structural characteristics.
Blinding	Blinding was not relevant to this study because the source of each of the mass spectrum collected in the experiments was needed to be conserved for downstream peak assignments and structure modeling.

Reporting for specific materials, systems and methods

We require information from authors about some types of materials, experimental systems and methods used in many studies. Here, indicate whether each material, system or method listed is relevant to your study. If you are not sure if a list item applies to your research, read the appropriate section before selecting a response.

Materials & experimental systems

n/a	Involved in the study
☐	☒ Antibodies
☐	☒ Eukaryotic cell lines
☒	☐ Palaeontology and archaeology
☒	☐ Animals and other organisms
☒	☐ Clinical data
☒	☐ Dual use research of concern
☒	☐ Plants

Methods

n/a	Involved in the study
☒	☐ ChIP-seq
☒	☐ Flow cytometry
☒	☐ MRI-based neuroimaging

Antibodies

Antibodies used

Secondary antibodies:
Anti mouse IgG (whole molecule) – HRP (produced in Goat) A4416 P code 1003321740, Source SLCK7186, Sigma Aldrich

Primary antibodies:

HEXIM1 Rabbit polyclonal IgG Catalog number 15676-1-AP, Lot number 00006924, Proteintech
Genbank accession number: BC006460
Immunogen catalog number: Ag8144
Gene ID (NCBI): 10614

CDK9 Rabbit polyclonal IgG 11705-1-AP, Lot 00047991, Proteintech
Genbank accession number: BC001968
Immunogen catalog number: Ag2318
Gene ID (NCBI): 1025
Cyclin T1 Rabbit polyclonal IgG 20992-1-AP, Lot 00016402 Proteintech
Genbank accession number:NM_001240
Gene ID (NCBI): 904

HSP90 13171-1-AP, Lot 00057547, Proteintech
Genbank accession number: BC023006
Gene ID (NCBI): 3320

Validation

All primary antibodies were sourced from verified suppliers, and each primary antibody was validated for use by the manufacturer. Detailed information, including validation tests and link to publications, is available on the suppliers website, accessed via the following links.

HEXIM1 Rabbit polyclonal IgG: https://www.ptglab.com/products/HEXIM1-Antibody-15676-1-AP.htm

CDK9 Rabbit polyclonal IgG: https://www.ptglab.com/products/CDK9-Antibody-11705-1-AP.htm

Cyclin T1 Rabbit polyclonal IgG: https://www.ptglab.com/products/CCNT1-Antibody-20992-1-AP.htm

HSP90 Rabbit polyclonal IgG: https://www.ptglab.com/products/HSP90-Antibody-13171-1-AP.htm

Eukaryotic cell lines

Policy information about cell lines and Sex and Gender in Research

Cell line source(s)

Two batches of commercial HeLa cell lines donated by members of the author's institution were used in this study.
Batch 1 was retrieved from passage no. 18, dated 12/09/12.
Batch 2 was donated on 01/10/2019

Authentication

None of the cell lines were authenticated

Mycoplasma contamination

Cell lines were not tested for mycoplasma contamination as this is not relevant to growing cells purely for protein extraction.

Commonly misidentified lines
(See ICLAC register)

N/A

Plants

Seed stocks	n/a
Novel plant genotypes	n/a
Authentication	n/a
